# UHPLC–MS/MS-Based Nontargeted Metabolomics Analysis Reveals Biomarkers Related to the Freshness of Chilled Chicken

**DOI:** 10.3390/foods9091326

**Published:** 2020-09-20

**Authors:** Tao Zhang, Shanshan Zhang, Lan Chen, Hao Ding, Pengfei Wu, Genxi Zhang, Kaizhou Xie, Guojun Dai, Jinyu Wang

**Affiliations:** 1 College of Animal Science and Technology, Yangzhou University, Yangzhou 225009, China; zhangt@yzu.edu.cn (T.Z.); zss18352764956@163.com (S.Z.); chenlan9326@163.com (L.C.); 15138343214@163.com (H.D.); wu_p_fei@163.com (P.W.); gxzhang@yzu.edu.cn (G.Z.); kzxie@yzu.edu.cn (K.X.); daigj@yzu.edu.cn (G.D.); 2 Joint International Research Laboratory of Agriculture and Agri-Product Safety, Ministry of Education, Yangzhou University, Yangzhou 225009, China

**Keywords:** chilled chicken, nontargeted metabolomics, ultra-high-performance liquid chromatography–mass spectrometry, metabolic biomarker, freshness

## Abstract

To identify metabolic biomarkers related to the freshness of chilled chicken, ultra-high-performance liquid chromatography–mass spectrometry (UHPLC–MS/MS) was used to obtain profiles of the metabolites present in chilled chicken stored for different lengths of time. Random forest regression analysis and stepwise multiple linear regression were used to identify key metabolic biomarkers related to the freshness of chilled chicken. A total of 265 differential metabolites were identified during storage of chilled chicken. Of these various metabolites, 37 were selected as potential biomarkers by random forest regression analysis. Receiver operating characteristic (ROC) curve analysis indicated that the biomarkers identified using random forest regression analysis showed a strong correlation with the freshness of chilled chicken. Subsequently, stepwise multiple linear regression analysis based on the biomarkers identified by using random forest regression analysis identified indole-3-carboxaldehyde, uridine monophosphate, s-phenylmercapturic acid, gluconic acid, tyramine, and Serylphenylalanine as key metabolic biomarkers. In conclusion, our study characterized the metabolic profiles of chilled chicken stored for different lengths of time and identified six key metabolic biomarkers related to the freshness of chilled chicken. These findings can contribute to a better understanding of the changes in the metabolic profiles of chilled chicken during storage and provide a basis for the further development of novel detection methods for the freshness of chilled chicken.

## 1. Introduction

Due to its advantages of high protein, low cholesterol and low fat, chicken is one of the most traded and consumed meats worldwide [1]. The yellow-feather chicken, a traditional breed in Asia, has a more distinctive flavor than many other commercial broilers [2]. However, in attempts to reduce ongoing outbreaks of animal influenza, particularly the avian influenza A (H7N9), live poultry markets are currently being restricted in most cities in China. In response, consumers are switching to buying fresh, chilled chicken through shops and supermarkets. As a result, the demand for chilled chicken has markedly increased, and the safety problems of chilled chicken have become a public health concern [3].

It is worth noting that, according to the European Parliament and Commission Regulation 178/2002, foods considered unsafe include not only those harmful to consumers, but also those unsuitable for human consumption [4]. From this perspective, unfresh and even spoiled chicken with poor appearance, taste and flavor are unsafe foods. Therefore, ensuring the freshness of chilled chicken is critical to public health.

The freshness of chicken decreases significantly with storage time—especially raw fresh chicken [1]. Decreasing chicken freshness is the result of spoilage and deterioration. In addition to lipid oxidation and autolytic enzymatic reactions, the spoilage of meat is considered to be the result of a variety of microbial activities [5]. The spoilage process caused by microbial activity produces a large number of low-molecular weight metabolites. The analysis and characterization of these metabolites can provide crucial information for meat control, classification and quality assessment [6,7]. Metabolomics can be defined as the comprehensive study of low-molecular weight (<1500 Da) metabolites in biologic cells, tissues, organs or organisms [8]. As one of the main omics tools, metabolomics has been widely used in screening biomarkers related to the freshness of eggs [9], fish [10,11], shellfish [12] and soybeans [13]. However, little research has been conducted on screening the freshness-related biomarkers of chilled chicken.

The main purpose of this study was to identify metabolic biomarkers related to the freshness of chilled chicken. To do this, we analyzed changes in metabolic profiles of chilled chicken induced by different lengths of storage time using an ultra-high-performance liquid chromatography–mass spectrometry (UHPLC–MS/MS)-based, nontargeted metabolomics approach. We then identified biomarkers related to the freshness (storage time) of chilled chicken by using random forest regression models and stepwise multiple linear regression methods. Our study provides a theoretical basis for developing new methods for rapid and accurate detecting of the freshness of chilled chicken.

## 2. Materials and Methods

### 2.1. Materials

Methanol (LCMS grade), acetonitrile (LCMS grade), ammonium acetate (LCMS grade), formic acid (LCMS grade) and ammonium hydroxide (LCMS grade) were purchased from CNW Technologies GmbH (Dusseldorf, Germany). The internal standard (2-chloro-L-phenylalanine, purity 98%) was purchased from Shanghai Hengbai Biotech C., Ltd., (Shanghai, China).

### 2.2. Sample Collection and Storage

Haiyang Yellow Chickens were collected from the farm of Jiangsu Jinghai Poultry Group co., LTD (Nantong, Jiangsu, China). Thirty-two chickens were randomly selected and slaughtered at 70 days of age. All chickens originated from the same farm, were the same age and were fed the same diet. The diet needs of Haiyang Yellow Chickens are shown in Table S1. The left breast muscle tissue of each chicken was removed from each carcass and placed in an individual sterile polythene bag. These samples were immediately transported to the laboratory in a car refrigerated at 4 °C. Once at the lab, all chicken breast muscles were divided into four groups (M1, M3, M5 and M7) under sterile conditions based on storage time (1 d, 3 d, 5 d and 7 d). Each group contained eight replicates weighing 50 g each. Finally, each sample was placed in a sterile polythene bag and stored at 4 °C. The entire process from slaughter to sample storage took less than one hour.

### 2.3. Metabolite Extraction

An approximately 50-mg sample was weighed and transported to a 1.5-mL Eppendorf microcentrifuge tube. After adding 1000 μL extract solvent (acetonitrile: methanol: water, 2:2:1, containing internal standard), the samples were vortexed for 30 s using a XW-80A vortex mixer (Kylin-Bell Lab Instruments Co., Ltd., Haimen, China), homogenized at 45 Hz for 4 min using a tissue grinding machine (JXFSTPRP-24, Shanghai, China) and sonicated for 5 min in an ice-water bath using an ultrasonic cell-crushing device (Fangao Microelectronics Co., Ltd., Shenzhen, China). The homogenate and sonicate circle was repeated three times, followed by incubation at −20 °C for 1 h and centrifugation at 12,000 rpm 4 °C for 15 min. The resulting supernatants were transferred to LC–MS vials and stored at −80 °C until the ultra-high-performance liquid chromatography–mass spectrometry (UHPLC–MS/MS) analysis. Quality control (QC) samples were prepared by mixing an equal aliquot of the supernatants from all of the samples.

### 2.4. Liquid Chromatography–Mass Spectrometry Analysis

LC–MS analysis was performed using a UHPLC system (1290, Agilent Technologies, Hilden, Germany) with a UPLC HSS T3 column (2.1 mm × 100 mm, 1.8 μm) coupled to a Q Exactive benchtop Orbitrap mass spectrometer (Orbitrap MS, Thermo, Waltham, USA). The mobile phase A was 0.1% formic acid in water for positive and 5-mmol/L ammonium acetate in water for negative; the mobile phase B was acetonitrile. The elution gradient was set as follows: 0 min, 1% B; 1 min, 1% B; 8 min, 99% B; 10 min, 99% B; 10.1 min, 1% B; 12 min, 1% B. The flow rate was 0.5 mL/min. The injection volume was 2 μL. The QE mass spectrometer was used for its ability to acquire MS/MS spectra on an information-dependent basis (IDA) during an LC/MS experiment. In this mode, the acquisition software (Xcalibur 4.0.27, Thermo) continuously evaluates the full scan survey MS data as it collects and triggers the acquisition of MS/MS spectra depending on the preselected criteria. ESI source conditions were set as follows: sheath gas flow rate—45 arb. unit; aux gas flow rate—15 arb. unit; capillary temperature—320 °C; full ms resolution—70,000; MS/MS resolution—17,500; collision energy—20/40/60 eV (NCE model); spray voltage—3.8 kV (positive) or 3.1 kV (negative).

### 2.5. Quality Control

Quality control (QC) samples were prepared by mixing an equal aliquot of the supernatants from all of the samples. The repeatability of the data and the stability of the instrument were assessed according to the overlapping base peak ion chromatograms (BPCs) of the QC samples. Residual material was determined by using the BPCs of blank samples. The reliability and stability of the instrument analysis were evaluated by using the principal component analysis (PCA) of all the samples [14].

### 2.6. Data Preprocessing and Annotation

MS raw data files were converted to the mzML format using ProteoWizard and processed by R package various forms of chromatography mass spectrometry (XCMS, version 3.2), including retention time alignment, peak detection and peak matching [15]. Retention time correction and peak detection were performed using the following parameters: method = “linear”, family = “gaussian”, plottype = “m”, bw = 10, minfrac = 0.5. Each sample was subsequently normalized to an internal standard of 2-chloro-L-phenylalanine [16]. Peaks with >50% missing values were excluded from the analysis. By default, any missing values were replaced by one half of the minimum value found in the dataset [17]. The preprocessing results generated a data matrix consisting of the retention time (Rt), mass-to-charge ratio (m/z) values and peak intensity. OSI-SMMS (version 1.0, Dalian Chem Data Solution Information Technology Co., Ltd., Dalian, China) was used for peak annotation after data processing with an in-house MS/MS database based on the secondary mass spectrometry data. An unsupervised dimensionality reduction principal component analysis (PCA) was used to describe the differences in metabolic profiles between different groups by using the R Project (http://www.r-project.org/, Vienna, Austria).

### 2.7. Differential Metabolites Analysis

The data were analyzed by SPSS version 22.0 for Windows (SPSS, Inc., Chicago, IL, USA). The Kruskal–Wallis test is a non-parametric statistical test that evaluates whether two or more samples are drawn from the same distribution [18]. It has been widely used in analyzing differences in the abundance of metabolites [19,20]. In our study, a nonparametric Kruskal–Wallis test was performed to compare the relative abundance of metabolites among different groups. Peaks with *p* < 0.05 were selected as differential metabolites. The heatmap clustering was analyzed and visualized by using the online Omicshare tool (https://www.omicshare.com/tools/, Guangzhou, China).

### 2.8. Random Forest Regression Analysis

Random forest regression analysis was performed by using the random forest package in R (https://CRAN.R-project.org/package=randomForest, Vienna, Austria). The importance of metabolites was evaluated by using the percent increase in mean square error (%IncMSE) and the increase in node purity (IncNodePurity). A ten-fold cross-validation was performed to identify metabolic biomarkers related to the freshness of chilled chicken. The performance of the random forest regression methods in identifying metabolic biomarkers was assessed using a receiver operating characteristic (ROC) curve analysis by the Anaconda distribution of Python (https://store.continuum.io/cshop/anaconda, Beaverton, USA). The profiles of final potential metabolic biomarkers were analyzed and visualized by using the ggplot2 package in R software. The KEGG pathway analysis was performed using online tool MetaboAnalyst 4.0 [21].

### 2.9. Stepwise Multivariate Linear Regression Analysis

Stepwise multiple linear regression model is an iterative algorithm, and it consists on adding and removing terms from a linear model based on their statistical significance in explaining the response value. The method begins with an initial model, and then compares the explanatory power (adjusted R^2^) of incrementally larger or smaller models [22]. The stepwise multiple linear regression analysis has been often applied to variable selection. In the present study, we used a stepwise multivariate linear regression analysis was used to identify key metabolic biomarkers related to the freshness of the chilled chicken based on the potential biomarkers screened by the random forest regression model using SPSS version 22.0 for Windows (SPSS, Inc., Chicago, IL, USA) [23]. Variables with a probability of *F*-test ≤ 0.05 entered the model and variables with a probability of *F*-test ≥ 0.10 were removed from the model. The adjusted coefficient of determination (R^2^) and *p* values were used to evaluate the goodness of fit of the constructed regression equations [15]. The independence and normal distribution of residuals were evaluated by the Durbin–Watson test and Quantile-Quantile (Q-Q) plot.

### 2.10. Pathway Enrichment of Potential Metabolic Biomarkers

To explore the metabolism pathways involved in chilled chicken spoilage, we performed a pathway-enrichment analysis of the 37 metabolic biomarkers using MetaboAnalyst 4.0. Parameters for Metabolic Pathway Analysis included normalization by sum and Pareto data scaling (mean-centered and divided by the square root of the standard deviation) of each variable presented. Small Molecule Pathway Database (SMPDB) metabolic pathways were utilized to determine the course of each metabolite.

## 3. Results and Discussion

### 3.1. Quality Control

**Figure 1:**
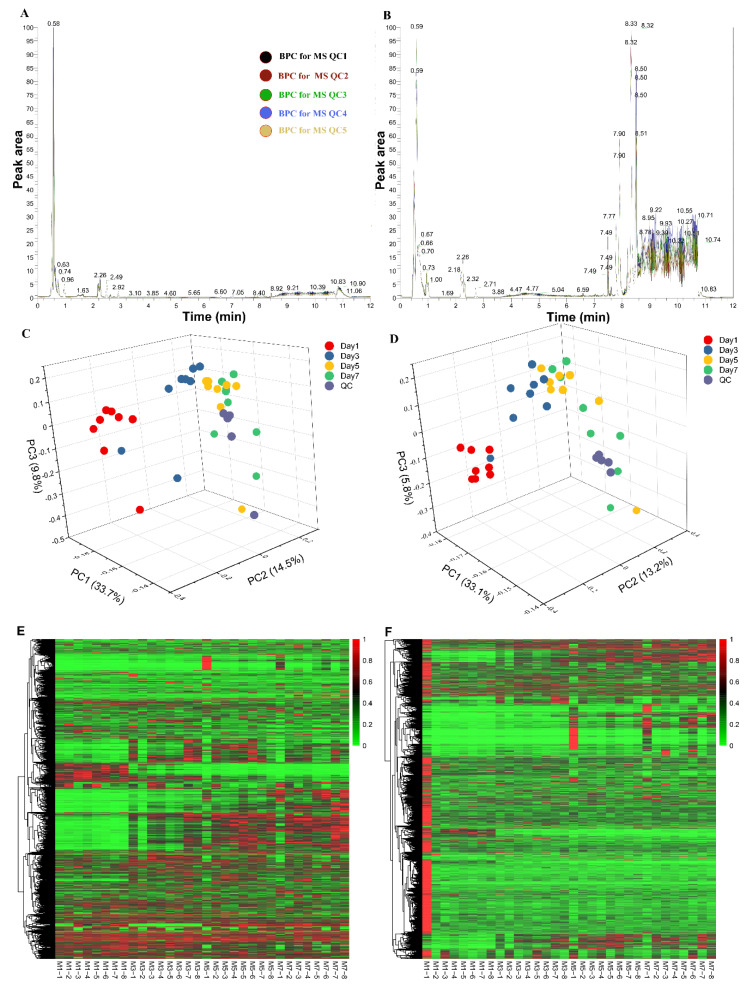
Quality control and metabolic profile analysis of chilled chicken samples. (**A**) Positive ion chromatograms of the five quality control samples; (**B**) negative ion chromatograms of the five quality control samples. Different colors represent samples; (**C**) principal component analysis (PCA) of all samples using positive ion mode data; (**D**) principal component analysis (PCA) of all the samples using negative ion mode data. PCA score plots displayed a low dispersion of quality control (QC) samples, which shows that there was minimal instrumental drift throughout the analysis; (**E**) heatmap clustering of all detected peaks using positive ion mode; (**F**) heatmap clustering of all the detected peaks using negative ion mode.

Base peak ion chromatograms (BPCs) were obtained based on the positive and negative ion mode. Different colors in Figure 1A,B indicate the overlapping BPCs of the five quality control samples. The horizontal coordinate represents the retention time; the vertical coordinate represents the relative abundance. The overlapping peaks indicated excellent repeatability of the extraction and detection of metabolites and high stability of the detection system [24]. PCA results of QC samples based on the metabolic profiles are shown in Figure 1C,D. The results were obtained based on the positive and negative ion mode data, respectively. PCA score plots displayed a low dispersion of QC samples, which shows that there is minimal instrumental drift throughout the analysis [9]. An analysis of blank samples showed that no significant peak was detected, indicating that there was no residual material or cross-contamination in the samples (Figure S1).

### 3.2. Metabolic Profiles Analysis of Chilled Chicken over Time

Meat spoilage caused by microorganisms results from microbial metabolism, leading to the production of molecules that alter the sensory quality of the products, in particular the aspect and odor [25]. Metabolomics has been defined as a field of research that involves the characterization, including identification and quantification, of the complete collection of small molecule metabolites in a biological system [19]. Thus, the characterization of molecule metabolites in spoilage process of chilled chicken using a metabolomic approach allows for a better control, evaluation, and understanding of meat spoilage [18].

One of the aims of this study was to analyze changes in metabolic profiles of chilled chicken induced by different lengths of storage time using LC–MS/MS-based nontargeted metabolomics approach. To this end, we characterized the metabolic profiles of all the 32 chilled chicken samples using the LC–MS/MS method. We found that the metabolic profiles were visibly different between fresh chicken (Day 1) and those that were stored for a longer time (Day 3, Day 5 and Day 7), as shown in the BPCs graphs in Figure S2. A total of 6147 and 6375 peaks were detected using the positive and negative ion modes, respectively. An integrated list of all 12,522 peaks is shown in Table S1. As shown in Figure 1E,F, all peaks were subjected to min–max normalization and cluster analysis. Heatmaps of all the peaks showed distinct hierarchical clustering of the samples by storage time.

**Figure 2:**
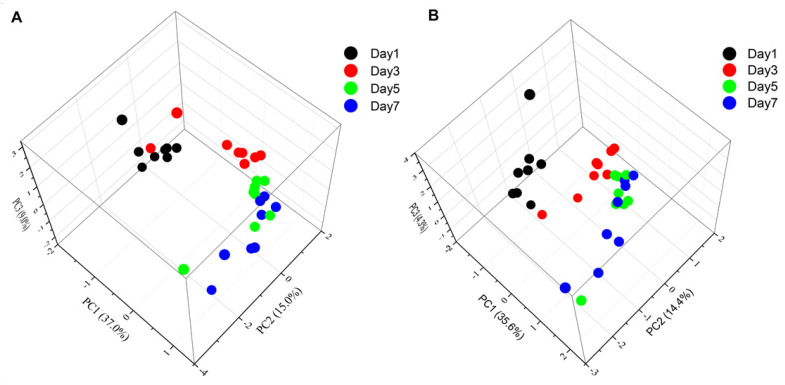
Principal component analysis (PCA) of the 32 samples based on metabolic peaks detected by positive ion mode (**A**) and negative ion mode (**B**). Different colors represent samples stored for different lengths of time.

PCA has been used to reveal changes in metabolic profiles of samples under different conditions [11,20]. In this study, we are very interested in the changes of metabolic profiles of chilled chicken induced by the length of storage time. We then performed a PCA analysis based on all the metabolic peaks across the 32 chicken samples except QC samples. The results of the PCA analysis on all the metabolic peaks across all 32 chicken samples showed differences among groups of chilled chicken stored for different lengths of time. The Day 1 group differed noticeably from the Day 3, Day 5 and Day 7 groups. The Day 3 group also differed from the Day 5 and Day 7 groups. However, the Day 5 group could not be distinguished from the Day 7 group (Figure 2). This seemed to indicate that difference in the length of storage time altered the metabolic profiles, and there was a significant change in the metabolic profiles of chilled chicken in the first five days of storage. From a metabolomic perspective, the chilled chicken meats in our study can be divided into two classes: “acceptable” (Days 1–4) and “unacceptable” (Days 5–7) based on the metabolic profiles of their different storage times, which is consistent with a previous study [26]. The above results indicate that the metabolomic method could be used to evaluate the freshness of chilled chicken.

### 3.3. Identification of Differential Metabolites

A total of 12,522 metabolic peaks were detected in chilled chicken samples using the LC–MS/MS method. For these metabolic peaks, attempts were made to annotate them using an in-house MS/MS database by OSI-SMMS. Consequently, 546 metabolites were successfully annotated based on the secondary mass spectrometry data (Table S2). To further screen metabolites related to the freshness of the chilled chicken, we identified differential metabolites based on the 546 annotated metabolites using Kruskal–Wallis test. A total of 265 differential metabolites were finally identified in our study (Table S3). Figure S3 shows the clustering heatmap of all the differential metabolites in samples stored for 1, 3, 5 and 7 days. The clustering heatmap displayed a consistent trend of the metabolic profiles with the PCA analysis.

### 3.4. Screening of Potential Biomarker Related to the Freshness of Chilled Chicken

**Figure 3:**
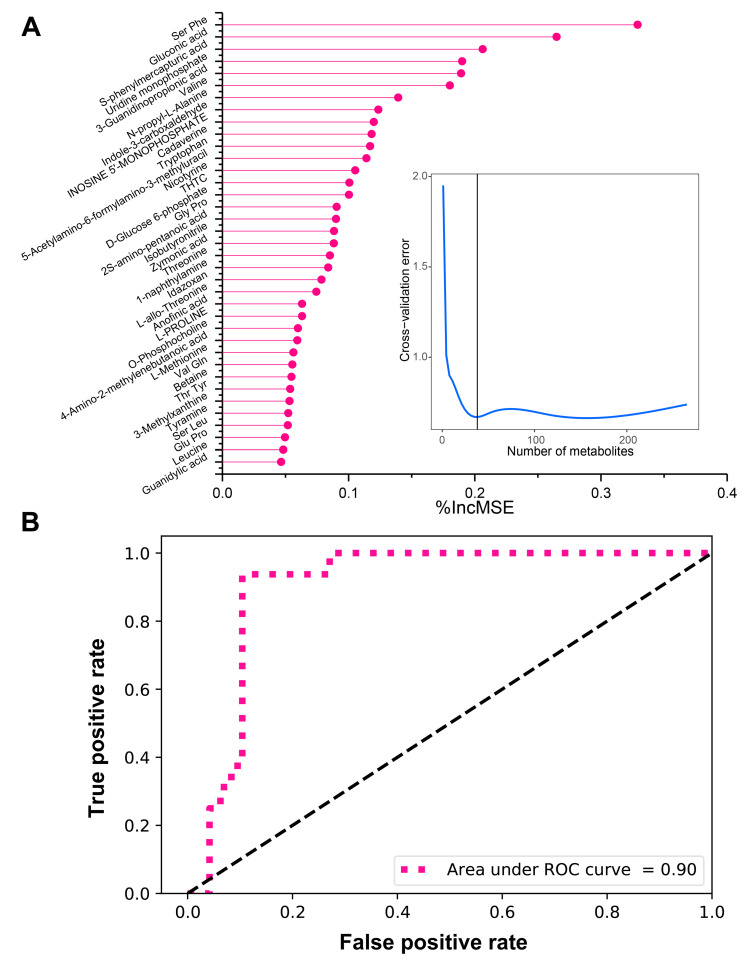
Potential biomarker screening by the random forest regression model. (**A**) Dot plot represents the top 37 metabolic biomarkers identified by using random forest regression. The metabolic biomarkers are ranked in descending order of the percent increase in mean square error (%IncMSE). The insert line plot represents 10-fold cross-validation with ten repeats that evaluates the importance of metabolites. The minimum cross-validation error was obtained when using 37 important metabolites; (**B**) receiver operating characteristic (ROC) curve of the potential metabolic biomarkers identified by the random forest regression model. The random forest model achieved a higher area under the ROC curve (AUC) of 0.90, indicating the excellent performance of the constructed model using the random forest method.

As a machine learning method, the random forest model is a regression tree technique that uses bootstrap aggregation and randomization of predictors to achieve a high degree of predictive accuracy [27]. The random forest model has been widely used to analyze complex metabolomics data [28,29,30,31,32,33] and has its unique advantages. First, it is relatively robust to outliers and noise. Second, it gives useful estimates of internal error, interaction and variable importance. Third, it has high prediction accuracy [34]. In the present study, we regressed the relative peak area of metabolites against the length of storage time of chilled chicken by using the random forest method. Table 1 shows the results of this 500-tree random forest analysis regression. Table S4 shows the importance of metabolites evaluated using the %IncMSE value. To determine the key metabolic biomarkers, we performed 10-fold cross-validation to analyze the relationship between the cross-validation error and the number of metabolites. Figure 3A shows that the cross-validation error decreased as the number of metabolites increased. The lowest cross-validation error was obtained when the number of metabolites was 37. We then fitted a parsimonious model based on these 37 metabolites to examine their predictive performance. Compared with the random forest model, the parsimonious model had a decreased mean of squared residuals and an increased percentage of variance explained, suggesting that it still achieved excellent predictive performance by using only the top 37 metabolites (Table 1). The above results indicated that the 37 metabolites with the highest %IncMSE value could be potential metabolic biomarkers related to the freshness of chilled chicken. The 37 potential metabolic biomarkers are shown in Table S5.

Receiver operating characteristic (ROC) curve analysis is a well-established method for assessing the performance of biomarkers. It is a graphic display which plots sensitivity estimates (probability of a true positive) against one minus specificity (probability of a false positive) of a marker for all possible threshold values. The performance of a marker is evaluated by the area under the ROC curve (AUC) in which a higher AUC value indicates a better marker performance [22]. In this study, we performed a time-dependent ROC analysis based on the 37 potential metabolic biomarkers. The performance of the potential biomarkers on predicting the freshness (storage time) of chilled chicken was evaluated by calculating the area under the ROC curve (AUC). An AUC value of one denotes that the performance is perfect, and an AUC value of 0.5 indicates random prediction performance [35]. The AUC value of the 37 potential biomarkers was 0.90, indicating the higher predictive performance of biomarkers identified by the random forest regression model (Figure 3B). In accordance with the present results, previous studies have demonstrated that the random forest method has been widely used in identifying biomarkers based on metabolic data and showed an excellent performance [36,37].

**Figure 4:**
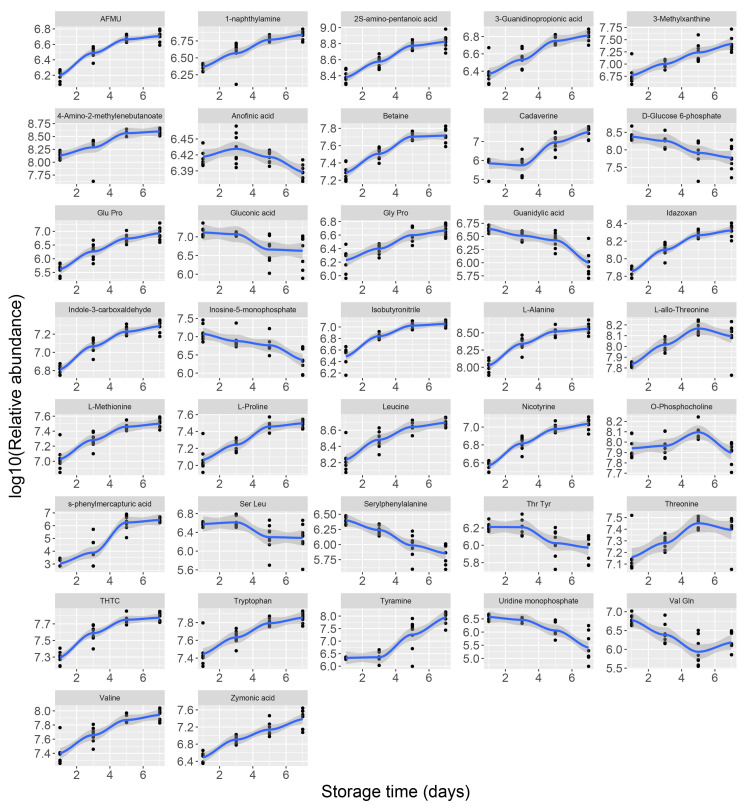
Profile analysis of potential biomarkers identified by using the random forest model.

In our study, 37 metabolites were identified as potential metabolic biomarkers related to the freshness of chilled chicken using the random forest regression method. We analyzed the profiles of the 37 potential metabolic biomarkers during storage over time. The results showed that 27 biomarkers were enriched while 10 biomarkers were depleted in the relative abundance in chilled chicken over time (Figure 4). The growth of spoilage microorganisms used the nutritional substrates of meat, produced metabolic byproducts, thereby caused the spoilage of meat [5]. We speculate that the depleted metabolites may be substrates used by the spoilage microorganisms. Moreover, the enriched metabolites may be metabolic byproducts produced during the spoilage of chilled chicken. Among the depleted metabolites, inosine 5′-monophosphate (IMP), guanidylic acid (GMP), gluconic acid and d-glucose-6-phosphate have been reported to be related to meat spoilage. Gluconic acid and d-glucose 6-phosphate are substrates used by meat spoilage bacteria during growth [5,38]. Inosine-5’-monophosphate and guanidylic acid are degradation products of nucleotides and have become important indicators to measure the freshness of meat [24]. In the enriched metabolites, tryptophan, cadaverine and tyramine have been reported to be key metabolites related to the freshness of chilled chicken [24]. These three metabolites belong to bioamines and their precursors. Bioamine content can indirectly reflect changes in bacterial content, it can also be used to measure food deterioration and shelf life [39].

**Figure 5:**
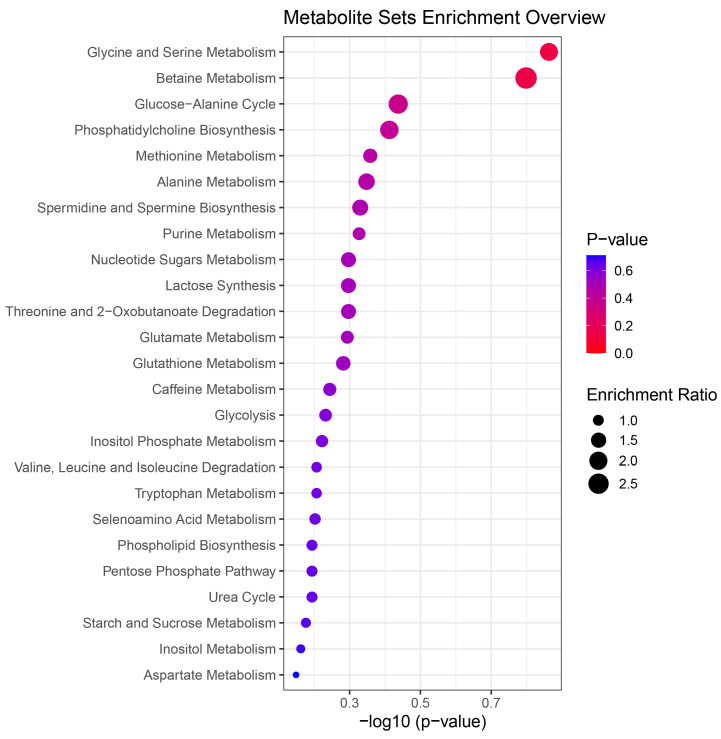
Pathway analysis of the 37 potential metabolic biomarkers. The node size is proportional to the enrichment ratio. The color from red to purple indicates *p* values from small to large.

In our pathway-enrichment analysis using MetaboAnalyst 4.0, we found that the 37 biomarkers were mainly involved in amino acid metabolism-related pathways such as the metabolism of glycine and serine, betaine, purine and methionine, suggesting the important roles of amino-acid metabolism-related pathways in chilled chicken spoilage (Figure 5). A previous study found that the purine metabolism pathway is involved in meat degradation [40]. In our study, inosinic acid, inosine and guanosine monophosphate were enriched in the purine metabolism pathway, indicating that these three metabolites may be metabolism products of chilled chicken spoilage and could be considered as metabolic biomarkers related to the freshness of chilled chicken.

### 3.5. Identification of Key Biomarkers Related to the Freshness of Chilled Chicken

**Figure 6:**
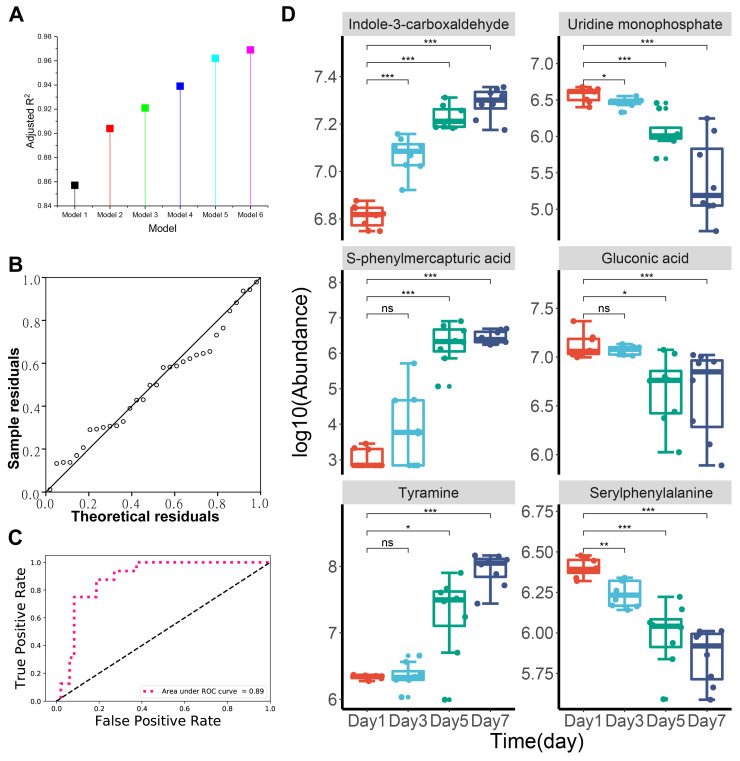
Key metabolic biomarkers selection using stepwise multiple linear regression model. (**A**) Adjusted R^2^ of the constructed six models. The adjusted R^2^ increased with the increase in the number of variables entered and Model 6 had the highest adjusted R^2^ of 0.969; (**B**) Q–Q plots for the residuals obtained from stepwise linear regression models; (**C**) combined ROC curve of the six key metabolic biomarkers. The six biomarkers achieved a combined AUC value of 0.89, indicating the high correlation of the six key biomarkers with the freshness of chilled chicken; (**D**) changes in the relative abundance of the six key biomarkers in chilled chicken during storage over time. A single asterisk indicates *p* < 0.05, double asterisks indicates *p* < 0.01, triple asterisks indicates *p* < 0.001 and ns indicates *p* > 0.05.

In the present study, to identify key metabolic biomarkers could be used as indicators for the freshness of chilled chicken, we performed a multiple linear regression analysis with stepwise variable selection based on the 37 potential metabolic biomarkers. Table 2 shows the significant models constructed by our stepwise multivariate linear regression analysis. We found that the adjusted R^2^ increased with an increase in the number of variables entered (Figure 6A) and all the adjusted R^2^ were greater than 0.85. The residuals showed both independence and normal distribution, as indicated by the Durbin–Watson statistic of 2.067 and Q–Q plot [38] (Table 2, Figure 6B). The above results suggest that the quality of the constructed models was quite high.

Model 6 had the maximum variables and the highest adjusted R^2^ (R^2^ = 0.969), indicating the best goodness of fit. Six key metabolic biomarkers were identified by Model 6: indole-3-carboxaldehyde (*β* = 1.97 × 10^−7^, *p =* 9.48 × 10^−7^), uridine monophosphate (*β* = −4.22 × 10^−7^, *p =* 1 × 10^−3^), s-phenylmercapturic acid (*β* = −3.37 × 10^−7^, *p =* 4.11 × 10^−6^), gluconic acid (*β* = 8.80 × 10^−8^, *p =* 8.62 × 10^−5^), tyramine (*β* = 1.26 × 10^−8^, *p =* 0) and Serylphenylalanine (*β* = −5.57 × 10^−7^, *p =* 1.60 × 10^−2^). Finally, we performed a combined ROC curve analysis to evaluate the predictive performance of the six key metabolic biomarkers selected by using the stepwise multivariate linear regression method (Figure 6C). The results show that even only using the six metabolic biomarkers, an excellent AUC value (0.89) was obtained, indicating the high correlation of the six metabolic biomarkers with the freshness (storage time) of the chilled chicken. Figure 6D shows the changes in the relative peak area (relative abundance) of the six metabolic biomarkers during storage of the chilled chicken over time.

Indole-3-carboxaldehyde (I3A)—also known as 3-formylindole or 3-indolealdehyde—belongs to the class of organic compounds known as indoles. It is a metabolite of indole-3-carbinol [16]. I3A has been detected in several different foods such as gram beans, brussels sprouts, cucumbers, cereals products and white cabbages. Therefore, I3A could be considered as a biomarker for the consumption of these foods [17,41]. Tyramine is a bioamine derived from the amino acid tyrosine. In foods, it is often produced by the decarboxylation of tyrosine during fermentation or decay [42]. Among all of the bioamines, tyramine poses the second highest potential threat to human health after histamine. A previous study has shown that the content of tyramine in chicken meat increased as storage days increased [24], which is consistent with our findings. We find that the relative content of tyramine is low during the first one to three days and increases significantly on Day 5 (*p* < 0.05), suggesting that tyramine could be an important biomarker related to the freshness of chicken. Gluconic acid is a substrate used by meat spoilage bacteria during growth and could be used as indicator for the freshness of chilled chicken [43]. To date, there have been no studies of s-phenylmercapturic acid, uridine monophosphate and Serylphenylalanine in association with meat freshness. Further research is needed to investigate their relationship with the freshness of chilled chicken in the future.

## 4. Conclusions

The present study provides new insights into the identification of metabolic biomarkers related to the freshness of chilled chicken. Thirty-seven potential metabolic biomarkers were screened using the random forest regression model. Based on the potential biomarkers identified by using the random forest regression model, we finally identified indole-3-carboxaldehyde, uridine monophosphate, s-phenylmercapturic acid, gluconic acid, tyramine and Serylphenylalanine as key metabolic biomarkers related to the freshness of chilled chicken. Our findings provide a comprehensive profile of the metabolites in chilled chicken during storage and provide possible biomarkers for developing new detection methods to determine the freshness of chilled chicken.

## Figures and Tables

**Table 1:** Parameters of the random forest model.

Model	N *^a^*	MSE *^d^*	VE (%) *^e^*
Random forest model *^b^*	500	0.86	81.98
Parsimonious model *^c^*	500	0.49	89.86

*^a^* number of trees; *^b^* random forest model fitted based on all the differential metabolites; *^c^* random forest model fitted based on the top 37 metabolites ranked by the percentage increase in mean square error (%IncMSE) value; *^d^* mean of squared residuals; *^e^* percentage of variance explained by the random forest model.

**Table 2:** Multivariate linear regression analysis of the 38 potential biomarkers.

Model	Variable	*Β ^b^*	*P ^c^*	Adjusted R^2^	Durbin–Watson
Model 1	Constant *^a^*	−1.324	4.00 × 10^−3^	0.857	2.067
	Indole-3-carboxaldehyde	3.87 × 10^−7^	1.99 × 10^−14^		
Model 2	Constant *^a^*	1.767	4.70 × 10^−2^	0.904	
	Indole-3-carboxaldehyde	2.56 × 10^−7^	7.06 × 1^−7^		
	Uridine monophosphate	−5.96 × 10^−7^	0		
Model 3	Constant *^a^*	2.014	1.50 × 10^−2^	0.921	
	Indole-3-carboxaldehyde	2.87 × 10^−7^	4.39 × 10^−8^		
	Uridine monophosphate	−7.47 × 10^−7^	2.41 × 10^−5^		
	S-phenylmercapturic acid	−2.30 × 10^−7^	1.20 × 10^−2^		
Model 4	Constant *^a^*	3.356	0	0.939	
	Indole-3-carboxaldehyde	2.46 × 10^−7^	3.07 × 10^−7^		
	Uridine monophosphate	−7.56 × 10^−7^	3.22 × 10^−6^		
	S-phenylmercapturic acid	−2.41 × 10^−7^	3.00 × 10^−3^		
	Gluconic acid	−7.87 × 10^−8^	5.00 × 10^−3^		
Model 5	Constant *^a^*	2.796	0	0.962	
	Indole-3-carboxaldehyde	2.37 × 10^−7^	1.20 × 10^−8^		
	Uridine monophosphate	−5.16 × 10^−7^	0		
	S-phenylmercapturic acid	−3.23 × 10^−7^	2.47 × 10^−5^		
	Gluconic acid	−9.42 × 10^−8^	0		
	Tyramine	1.27 × 10^−8^	0		
Model 6	Constant *^a^*	3.964	1.76 × 10^−5^	0.969	
	Indole-3-carboxaldehyde	1.97 × 10^−7^	9.48 × 10^−7^		
	Uridine monophosphate	−4.22 × 10^−7^	1.00 × 10^−3^		
	S-phenylmercapturic acid	−3.37 × 10^−7^	4.11 × 10^−6^		
	Gluconic acid	−8.80 × 10^−8^	8.62 × 10^−5^		
	Tyramine	1.26 × 10^−8^	0		
	Serylphenylalanine	−5.57 × 10^−7^	1.60 × 10^−2^		

*^a^* constant in each model; *^b^* significance level of each variable; *^c^* unstandardized regression coefficient.

## Supplementary Materials

The following are available online at https://www.mdpi.com/2304-8158/9/9/1326/s1, Table S1: The diet needs of Haiyang Yellow Chickens, Table S2: Integrated list of the 12,522 peaks detected by using the nontargeted metabolomics, Table S3: List of the 546 metabolites annotated based on the secondary spectrometry data, Table S4: Differential metabolites during storage of chilled chicken, Table S5: Potential biomarkers screened using the random forest method. Figure S1: Base peak ion chromatograms (BPCs) of blank samples, Figure S2: Base peak ion chromatograms (BPCs) of chilled chicken samples at different storage time, Figure S3: Heatmap clustering of the differential metabolites.
