# Supplementary material for: UHPLC–MS/MS-Based Nontargeted Metabolomics Analysis Reveals Biomarkers Related to the Freshness of Chilled Chicken
Source: Foods. 2020 Sep 20;9(9):1326. doi: 10.3390/foods9091326 (PMC7555583; doi:10.3390/foods9091326)
Supplement: Supplementary file 1 [file foods-09-01326-s001.zip › Supplementary Materials.docx]

**Table S1. Diet needs of Haiyang Yellow Chicken**

| Nutritional components | 1-3 weeks of age | 4-7 weeks of age | 8-10 weeks of age |
| --- | --- | --- | --- |
| Metabolizable energy (kcal/kg) | 2900 | 2950 | 3000 |
| Protein (%) | 20.00 | 18.00 | 16.00 |
| Ca (%) | 1.00 | 1.00 | 1.00 |
| Available potassium (%) | 0.47 | 0.45 | 0.42 |
| Methionine (%) | 0.50 | 0.40 | 0.33 |
| Methionine & cysteine (%) | 0.82 | 0.65 | 0.58 |
| Lysine (%) | 1.10 | 0.90 | 0.80 |
| Threonine (%) | 0.76 | 0.74 | 0.68 |
| Tryptophan (%) | 0.19 | 0.18 | 0.16 |
| Arginine (%) | 1.19 | 1.10 | 1.00 |
| Leucine (%) | 1.15 | 1.09 | 0.93 |
| Isoleucine (%) | 0.76 | 0.73 | 0.62 |
| Phenylalanine (%) | 0.69 | 0.65 | 0.56 |
| Phenylalanine & Tyrosine (%) | 1.28 | 1.22 | 1.00 |
| Histidine (%) | 0.33 | 0.32 | 0.27 |
| Proline (%) | 0.57 | 0.55 | 0.46 |
| Valine (%) | 0.86 | 0.82 | 0.70 |
| Glycine & Serine (%) | 1.19 | 1.14 | 0.97 |
| Na (%) | 1.00 | 0.90 | 0.80 |
| Cl (%) | 0.15 | 0.15 | 0.15 |
| Fe (mg/kg) | 80 | 80 | 80 |
| Cu (mg/kg) | 8 | 8 | 8 |
| Mn (mg/kg) | 80 | 80 | 80 |
| Zn (mg/kg) | 60 | 60 | 60 |
| I (mg/kg) | 0.35 | 0.35 | 0.35 |
| Se (mg/kg) | 0.15 | 0.15 | 0.15 |
| Linoleic acid (%) | 1 | 1 | 1 |
| Vitamin A (IU/kg) | 5000 | 5000 | 5000 |
| Vitamin D (IU/kg) | 1000 | 1000 | 1000 |
| Vitamin E (IU/kg) | 10 | 10 | 10 |
| Vitamin K (mg/kg) | 0.50 | 0.50 | 0.50 |
| Thiamin (mg/kg) | 1.80 | 1.80 | 1.80 |
| Riboflavin (mg/kg) | 3.60 | 3.60 | 3.00 |
| Pantothenic acid (mg/kg) | 10 | 10 | 10 |
| Niacin (mg/kg) | 35 | 30 | 25 |
| Pyridoxine (mg/kg) | 3.50 | 3.50 | 3.00 |
| Biotin (mg/kg) | 0.15 | 0.15 | 0.15 |
| Folic Acid (mg/kg) | 0.55 | 0.55 | 0.55 |
| Vitamin B_12_ (mg/kg) | 0.01 | 0.01 | 0.01 |
| Choline (mg/kg) | 1000 | 750 | 500 |

**Table S2.** Integrated list of the 12,522 peaks detected by using the nontargeted metabolomics

**Table S3.** List of the 546 metabolites annotated based on the secondary spectrometry data

**Table S4.** Differential metabolites during storage of chilled chicken

**Table S5.** Potential biomarkers screened using the random forest method


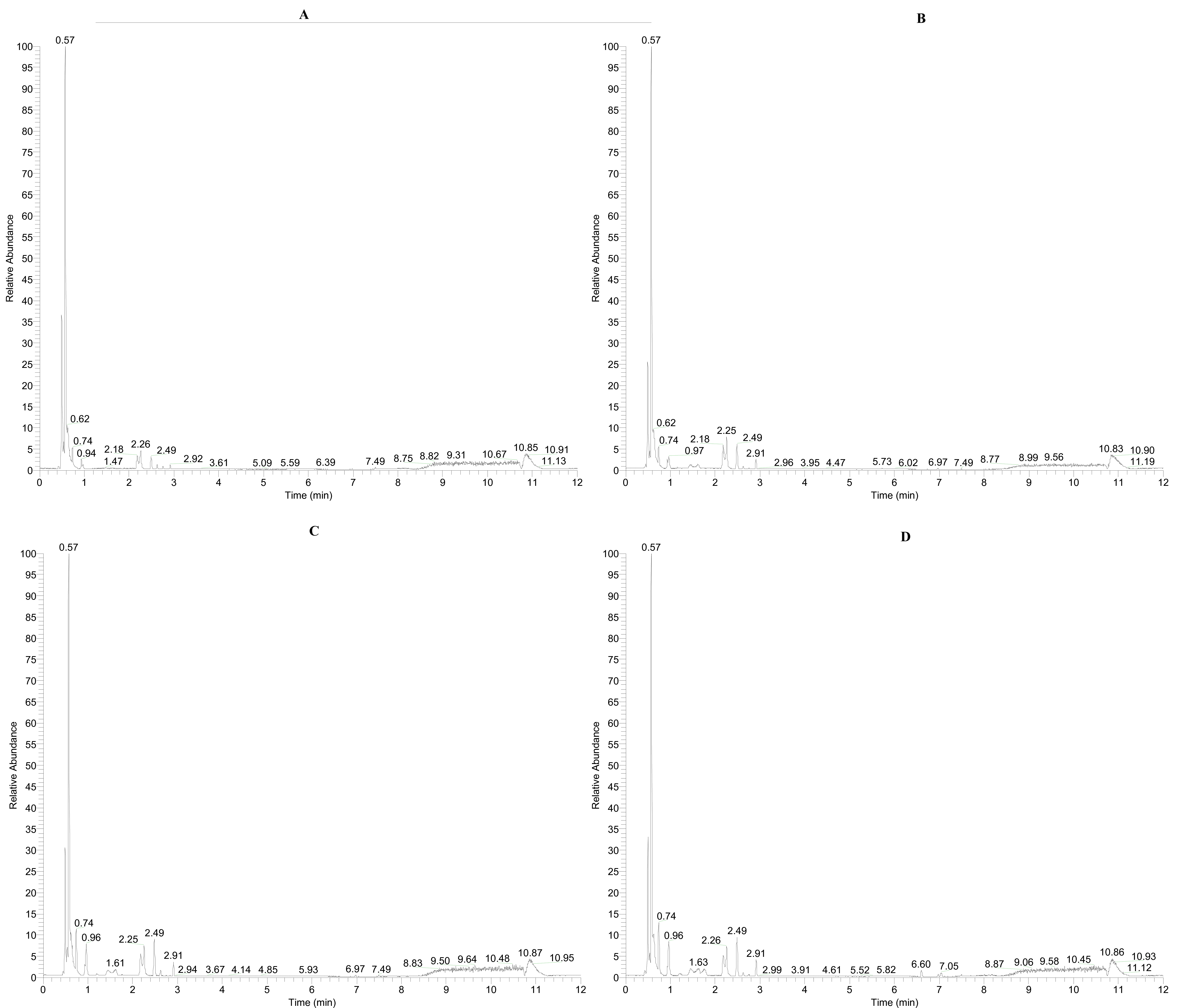


**Figure S1.** Base peak ion chromatograms (BPCs) of blank samples


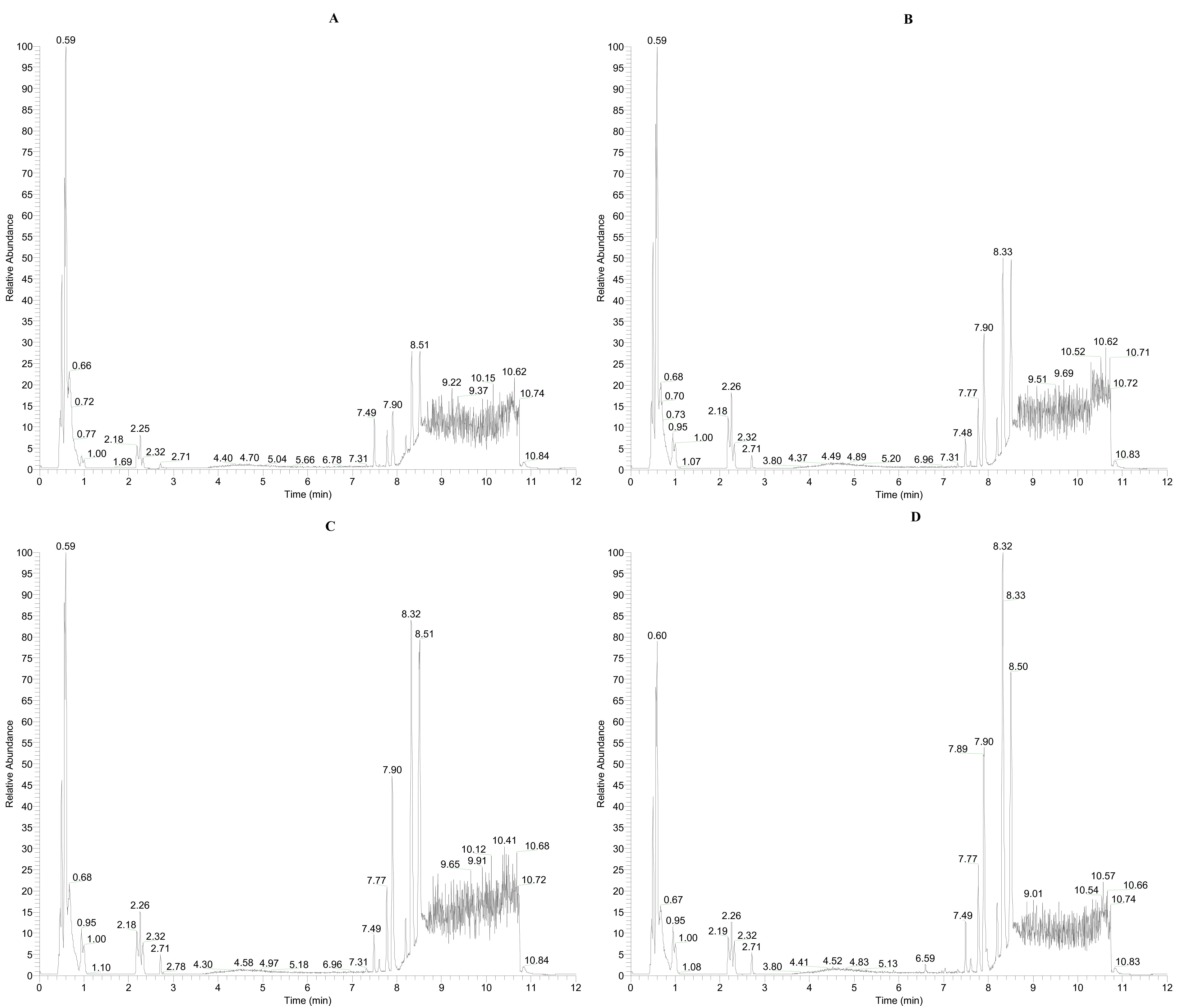


**Figure S2.** Base peak ion chromatograms (BPCs) of chilled chicken samples at different storage time


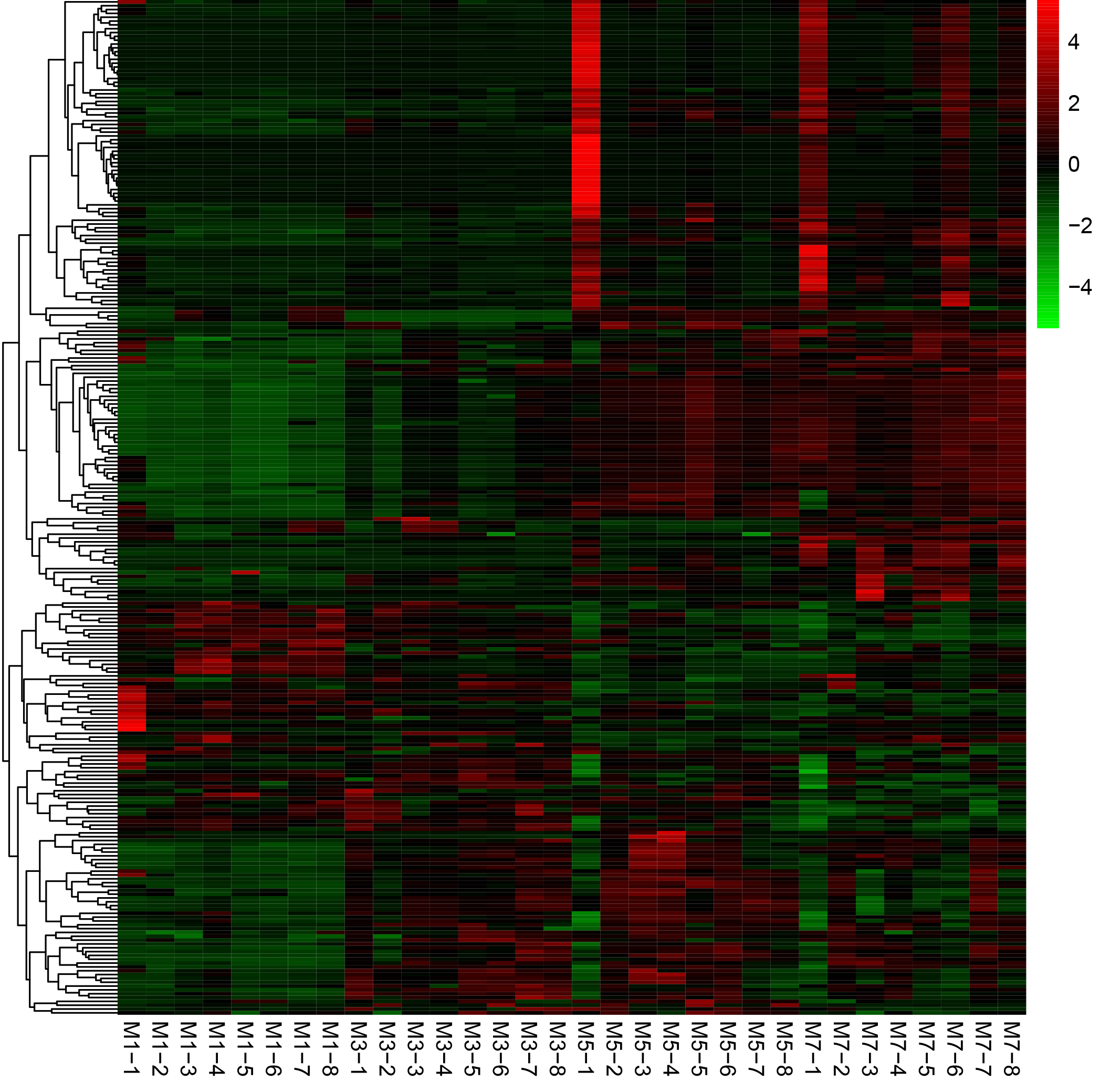


**Figure S3.** Heatmap clustering of the differential metabolites.
