# Supplementary figures and images for: UHPLC–MS/MS-Based Nontargeted Metabolomics Analysis Reveals Biomarkers Related to the Freshness of Chilled Chicken
Source: Foods. 2020 Sep 20;9(9):1326. doi: 10.3390/foods9091326 (PMC7555583; doi:10.3390/foods9091326)

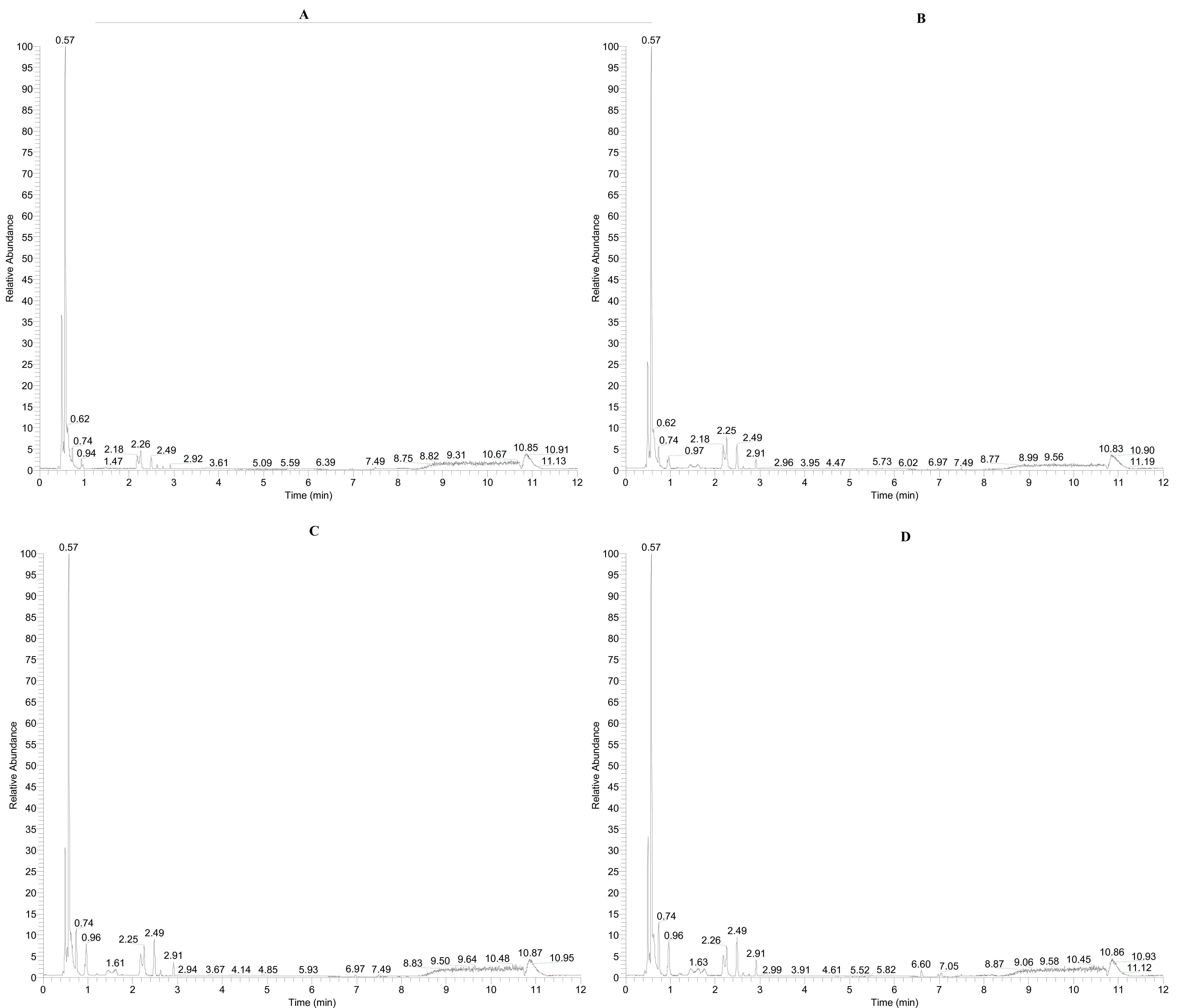

Supplement: Supplementary file 1 [file foods-09-01326-s001.zip › Figure S1.tif]

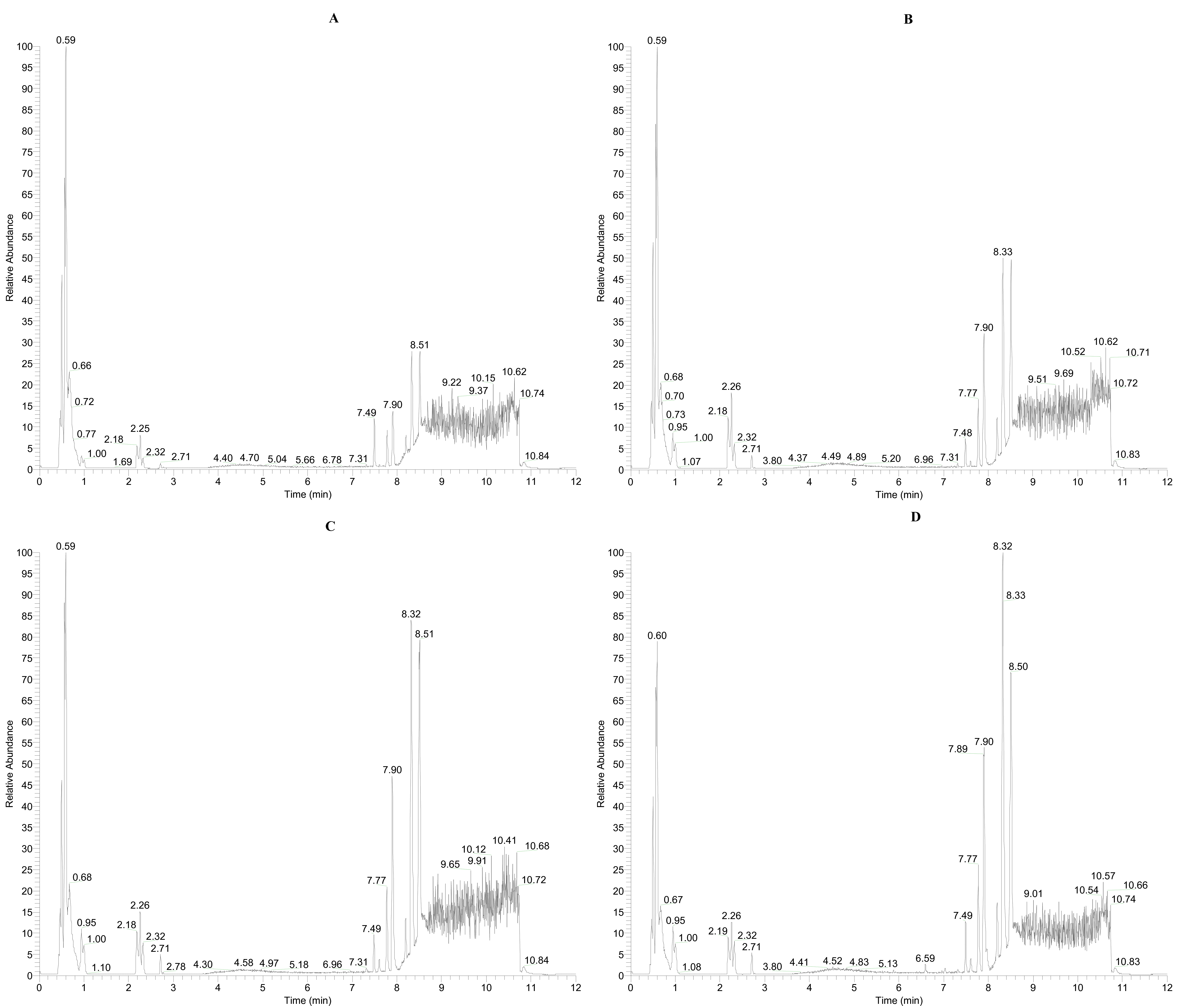

Supplement: Supplementary file 1 [file foods-09-01326-s001.zip › Figure S2.tif]

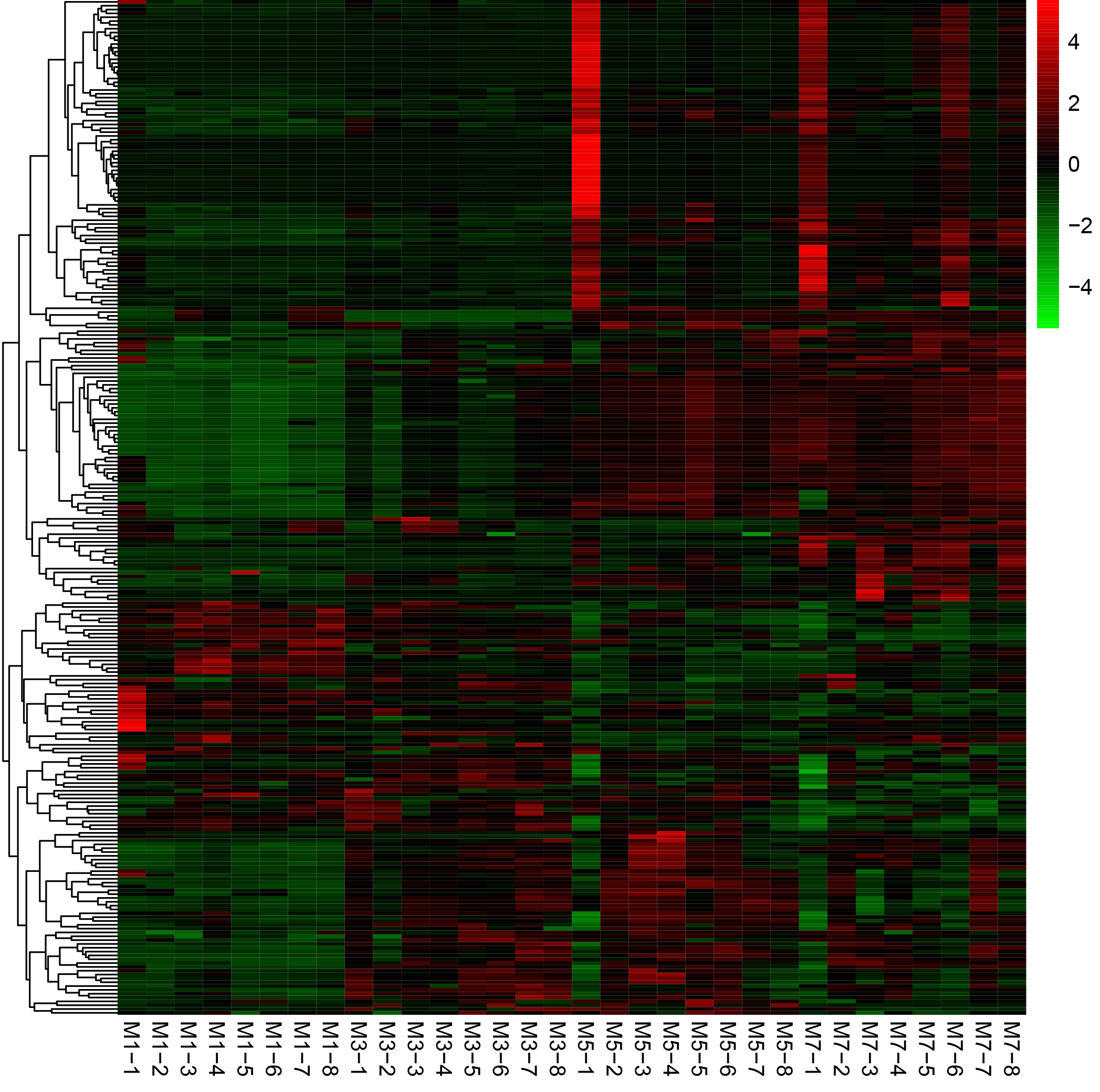

Supplement: Supplementary file 1 [file foods-09-01326-s001.zip › Figure S3.tif]
